# AMD Genetics in India: The Missing Links

**DOI:** 10.3389/fnagi.2016.00115

**Published:** 2016-05-23

**Authors:** Akshay Anand, Kaushal Sharma, Suresh K. Sharma, Ramandeep Singh, Neel K. Sharma, Keshava Prasad

**Affiliations:** ^1^Neuroscience Research Lab, Department of Neurology, Post Graduate Institute of Medical Education and Research Chandigarh, India; ^2^Centre for Systems Biology and Bioinformatics, Panjab UniversityChandigarh, India; ^3^Department of Statistics, Panjab UniversityChandigarh, India; ^4^Advanced Eye Centre, Post Graduate Institute of Medical Education and ResearchChandigarh, India; ^5^Neurobiology Neurodegeneration and Repair Laboratory, National Eye InstituteBethesda, MD, USA; ^6^Institute of BioinformaticsBangalore, India; ^7^YU-IOB Center for Systems Biology and Molecular Medicine, Yenepoya UniversityMangalore, India; ^8^NIMHANS-IOB Proteomics and Bioinformatics Laboratory, Neurobiology Research Centre, National Institute of Mental Health and NeurosciencesBangalore, India

**Keywords:** age related macular degeneration, SNP, biomarkers, longitudinal analysis, snSNPs, bio-informatics analysis, statistical modeling, Mendelian randomization

## Abstract

Age related macular degeneration is a disease which occurs in aged individuals. There are various changes that occur at the cellular, molecular and physiological level with advancing age (Samiec et al., 1988; Sharma K. et al., 2014). Drusen deposition between retinal pigment epithelium (RPE) and Bruch’s membrane (BM) is one of the key features in AMD patients (Mullins et al., 2000; Hageman et al., 2001) similar to Aβ/tau aggregates in Alzheimer’s disease (AD) patients. The primary goal of this review is to discuss whether the various candidate genes and associated biomarkers, that are known to play an independent role in progression of AMD, exert deleterious effect on phenotype, alone or in combination, in Indian AMD patients from the same ethnic group and the significance of such research. A statistical model for probable interaction between genes could be derived from such analysis. Therefore, one can use multiple modalities to identify and enrol AMD patients based on established clinical criteria and examine the risk factors to determine if these genes are associated with risk factors, biomarkers or disease by Mendelian randomization. Similarly, there are large numbers of single nucleotide polymorphisms (SNPs) identified in human population. Even non-synonymous SNPs (nsSNPs) are believed to induce deleterious effects on the functionality of various proteins. The study of such snSNPs could provide a better genetic insight for diverse phenotypes of AMD patients, predicting significant risk factors for the disease in Indian population. Therefore, the prediction of biological effect of nsSNPs in the candidate genes and the associated grant applications in the subject are highly solicited.Therefore, genotyping and levels of protein expression of various genes would provide wider canvas in genetic complexity of AMD pathology which should be evaluated by valid statistical and bioinformatics’ tools. Longitudinal follow up of Indian AMD patients to evaluate the temporal effect of SNPs and biomarkers on progression of disease would provide a unique strategy in the field.

## Introduction

Diseases associated with aging are being reported with increasing frequency due to enhanced human lifespan and/or risk factors. Aging-associated diseases include arthritis, dementia, Alzheimer’s disease (AD), AMD and cataract. The preclinical studies on AMD and its treatment strategies are lacking due to nonavailability of well characterized animal models as well as absence of population based genetic epidemiological studies. Mice lacking with monocyte chemoattractant protein-1 (*CCL-2*) and its receptor *CCR-2* was found to develop the cardinal feature of AMD in mouse retina (Ambati et al., 2003). Recently, several risk genes for AMD have been discovered by examining the DNA samples from Caucasian (white with European ancestry) subjects and have been found to be associated with *CCL-2* and complement factor H (*CFH*) polymorphisms. Other genes are also being investigated in AMD pathogenesis in various populations, some with conflicting and unverified reports, others which are validated, however, equally large comprehensive genetic studies from India, particularly from North-West India remain limited thus impacting India’s reduced role in pioneering AMD diagnostics and therapies. It would be advisable to establish a population based genetic profile database of Indian AMD patients to dissect the role of specific genetic factors involved in pathogenesis of AMD. This could be achieved by examining AMD patients for major risk single nucleotide polymorphisms (SNPs), biomarkers and risk factors including follow up of AMD patients at the interval of 1 year for investigating SNP-phenotype correlation as well as temporal association of biomarkers with disease progression. The changes in SNPs of genes could be analyzed for their impact on disease pathology by their changes in structural and functional aspect by informatics approach. Thus, it will provide an insight of how the changes at genomic level reflect at the protein level, affecting protein function and contributing towards the progression of AMD.

Briefly, the primary aim of this review is to discuss the role of allelic variations among various genes and also to argue their nature and causal effect on protein expression, AMD phenotype and progression. Moreover, it is also to describe the need to examine the role of socio-demographic and risk modifying factors specific to Indian population in the pathogenesis of AMD so that a well characterized AMD population database is created for future drug targeting and clinical trials.

As a natural consequence of above cited literature in Table 1, we emphasize the need to describe the interaction between various gene’s SNPs and biomarkers across various loci in AMD patients that could be causal to and prognostication of AMD progression in Indian population. Moreover, identification of the genetic susceptibility from population based studies could be further encapsulated by proposing a statistical model which can provide means to strategize public health initiatives through new prevention and treatment schemes for those suspected of suffering from AMD. Additionally, the conclusion of genetic data could be strengthened by including a longitudinal follow up of AMD patients in order to advance our understanding of the effect of SNPs and biomarkers along the course of disease, examine the clinico-pathological and etiological relevance of these observations and to investigate whether these alterations are a “cause” or a “consequence” of AMD. To further evaluate how these SNPs lead to structural and functional abnormalities in the protein, *in silico* studies would be required to confirm the nature of SNPs by using standard bio-informatics tools. The results of such bioinformatics analysis can provide a biological annotation of nsSNP in the candidate genes. This can predict the impact of variation in structure and function of proteins. Disease risk can also be predicted based on effect of nsSNPs on the function of protein in the early age of the patients who will likely to have AMD in the later stage of their life.

**Table 1:** **The overview of Indian AMD investigations carried out in India showing various risk loci that have neither been examined collectively in one set of patients nor analyzed for SNPs**.

	Study design	Age range	Region	Diagnostic criteria used	Subjects/Type of controls	Sample size	Alleles or SNPs	Other genes	Reference
1	Case control of TIMP3	68.8 ± 3.1 years for wet AMD, 64.4 ± 4.8 years for dry AMD	South India	AREDS	AMD/Normal control	250	rs713685 rs6518799 rs743751	*FBLN6* *FBLN5* *MMP2* *MMP1* *MMP3* *DCN* *LUM* *EPYC* *TIMP2* *MMP9*	Kaur et al. (2010)
2	Case control of CFH	68.8 ± 3.1 years for wet AMD, 64.4 ± 4.8 years for dry AMD	South India	AREDS	AMD/Normal control	250	rs1061170	*LOC387715* *HTRA1*	Kaur et al. (2008)
3	Case control of CFH	62.4 ± 10.2 for early AMD, 69.2 ± 7.7 for late AMD	South India	AREDS	AMD/Normal control	100	rs1061170 rs3766404 rs3753394 rs800292 rs3753396 rs1065489	*TLR4, APOE*	Kaur et al. (2006)
4	Case control study VEGF	–	South India	–	Diabetic Retinopathy/diabetic control	120	Promoter region	*Nitric oxide*	Suganthalakshmi et al. (2006)
5	Case control study for Ccl2 and Ccr2	66.56	North India	Age and diagnosis for AMD	AMD/normal control	133	rs4586 (CCL2), rs1799865 (CCR2)	–	Anand et al. (2012)
6	Case control study for ARSM2 and CFH	60	South India	Wisconsin Age-Related Maculopathy Grading System	AMD/disease control	3569	rs1061170 for CFH rs10490924, rs2672598, rs10490923 for ARSM2	HTRA1, C2, CFB	Sundaresan et al. (2012)
7	Case control study for VEGFR2	66.56	North India	Age and diagnosis for AMD	AMD/normal control	115	rs1531289 and rs2305948 for VEGFR2		Sharma et al. (2012)
8	Case control study for CFH	66.56	North India	Age and diagnosis for AMD	AMD/normal control	115	rs1061170 for CFH		Sharma et al. (2013)
9	Case control study for TLR-3	66.5	North India	Age and diagnosis for AMD	AMD and normal subjects	115 for *TLR3*	rs3775291		Sharma et al. (2014)

The integrative approach including statistics and bioinformatics can deal with heterogenic complexity of AMD genetics. In genome-wide association study (GWAS), missing genetic links and implication of variants found in untranslated region of the genome could be annotated by bioinformatics analysis and could also predict the probable interaction between various associated genes in disease pathology. Moreover, the effect of environmental factors on genetic variants could be correlated with Mendelian randomization approach or by Sequential Kernel Association Test (SKAT) analysis. Hence, the integrative approach in AMD genetics could enhance the productivity and better translational benefit in such studies.

## Key Questions Need to be Addressed in Indian AMD Genetic Studies

AMD is a degenerative disease of eye with irreversible central vision loss in old age. There is no reliable treatment and diagnostic or prognostic biomarkers unique to Indian population. Therefore, we must have to drive such genetic studies which may result not only in the discovery of new biomarkers for validation of new therapies and monitoring treatment outcomes but also investigating the role of SNPs in disease prognosis. Such studies will also resolve the conflicting reports on the association of various loci, candidate genes and associated SNPs by examining them in the same population.

Also, the following key questions need to be addressed in Indian AMD scenario:

- Is there any one or set of novel SNPs or biomarkers causal to Indian AMD?
- What is the expression profile of such biomarkers analyzed at the certain time intervals by recruiting Grade 3 (AREDS) AMD patients and if these are causally related to the disease progression?
- Is there any gene-demography or SNP-protein association which can be linked to AMD by either a predictive model for probable interaction between genes or by Mendelian randomization or bioinformatics approach?
- Do the SNPs or nsSNPs result in changes in secondary and tertiary structures of proteins coded thus impacting their function?
- Do the risk factors like diet, smoking, alcohol consumption, co-morbidity etc influence the molecular progression or severity of the disease?

## Complex Genetic Network in AMD Pathology

AMD is a third most devastating eye disorder which is characterized by irreversible and incurable hampered vision due to degeneration of macula affecting more than 50 million elderly people in the world. Macula consists of high density of photoreceptors which helps in central and sharp vision. AMD is prevalent in about 2% of people over the age of 50 which rises about 8% in >65 years old and 20% of those over 85 years of age having this condition.

AMD can be categorized into dry and wet forms. Drusen deposition in between the retinal pigment epithelium (RPE) and choroids is a characteristics feature of dry AMD. The abnormal neovascularization of choroids penetrate the RPE and disrupt its integrity. These abnormal choroidal blood vessels are leaky in the nature therefore leads to development of wet AMD.

AMD literature is replete with both confirmatory and conflicting reports of over 19 major genes and their SNPs which have been postulated to be associated with AMD but with very limited large studies examining all the alleles in the same study thus hampering its translation into clinical application. We provide here one example of our experience with another neurodegenerative disorder in order to highlight the value of gene demography investigations in Indian scenario. Our study on Parkinson’s Disease (PD) patients in North-West region of India revealed 40% *PARK2* mutation screened by single strand conformation polymorphism (SSCP) in these patients which had never been exposed with smoking. We compared the demographic and community data of other neurological patients with PD patients, found Sikh and female patients from rural background exhibit significantly higher *PARK2* mutation as compared to urban and male population. We also examined the age of onset of PD in rural background which was significantly lower as compared to urban background PD patients (Prabhakar et al., 2010; Vinish et al., 2010). Therefore, the inclusion of alleles or SNPs-demographic association studies a large set of population could assist to examine the demographic parameters with disease progression that are relevant to Indian scenario.

The environmental factors share the causal effect along with genetic factors in AMD pathophysiology. Environmental factors include smoking, high cholesterol diet, carotinoid, Vitamin A, Vitamin E, zinc, age, sex *etc*.

Several environmental factors have been reported for AMD progression which includes cigarette smoking, (Seddon et al., 1996) higher body mass index (BMI; Seddon et al., 2003), and dietary carotenoids (van Leeuwen et al., 2005). Human hepatic lipase is one of the important lipase and plays an important role in lipid metabolism which convert intermediate-density lipoprotein to low-density lipoprotein. It is abundantly expressed in hepatic cells and adrenal gland. Polymorphism of *LIPC* has shown strong evidence for their relation with pathogenesis of AMD. Seddon et al. (2010) have conducted GWAS and found protective effect of TT genotype vs. CC genotype. It further revealed that SNP variation at promoter region (rs1046817) which influence the *LIPC* expression, have showed strong association with AMD pathogenesis independent to demographic and environmental factors. Recently, Neale et al. (2010) revealed the *LIPC* variance rs493258 and its correlation with AMD pathology, and also demonstrated that *LIPC* variances rs493258 along with rs10468017 strong association with advanced AMD progression by influencing the HDL levels.

Tissue inhibitor of metalloproeinase-3 (*TIMP-3*) is another known regulator of the functioning of metalloproteinases and can degrade the extracellular matrix (ECM). *TIMP-3* plays an imperative in maintaining the ECM and has been found to be anti-angiogenic. In a genome wide association study conducted by Chen and colleagues, they identified a susceptibility locus in AMD pathology near *TIMP3*. Additionally, they have also revealed the strong association with *LIPC* polymorphism in the same set population in AMD patients (Chen et al., 2010). In an Indian study Kaur et al. (2010) has found that a SNP identified at ≈100 kb upstream of *TIMP-3* was associated with AMD.

*TIMP3* is an ECM protease and has been found to be anti angiogenic (Sang, 1998). In a GWAS, Chen et al. (2010) along with another separate study it has been found that a SNP present at ≈100 kb upstream of *TIMP-3* raises the vulnerability to AMD in diverse populations. It has been suggested that elevated level of *TIMP-3* in eyes in AMD results in thickening of Bruch’s membrane (BM; Kamei and Hollyfield, 1999), and recent study on human RPE has shown that *TIMP-3* is one of the strongest candidate gene in AMD pathophysiology (Strunnikova et al., 2010).

Immediate Early Response-3 (*IER-3*) is another regulatory protein involved in apoptosis mediated by NF-κB pathways. The expression levels of *IER-3* could be enhanced by various cellular responses, viral infection, and with interaction with cytokines. The *IER-3* was also found to be involved in systemic immunity and in cardiovascular system. The knockout study revealed that *IER-3* deficiency could hamper the immunity, inflammation and genomic stability (Arlt and Schäfer, 2011). This group has also found the effect of *IER3* which interacts with various signaling network specifically *NF-kB, MAPK/ERK* and *PI3K/Akt* and have been found to have abnormal immune function and increased inflammation with hypertension and impaired in genomic stability (Sasada et al., 2008). Moreover *IER-3* has also been found to be upregulated in apoptotic processes in Sezary (SzS) which was mediated by TNF-α in patient lymphocytes which might be linked to its pathogenesis. Akilov et al. (2012) investigated that CD4^+^ (CD26) lymphocytes of SzS found *IER-3* upregulation, with low level of intracellular reactive oxygen species (ROS) and reduced *TNFR1* expression in patients. Steensma et al. (2009) described have shown the IER-3 genes was translocated in the patients of myelodysplastic syndrome (MDS), markedly decreasing expression.

On the other hand, Vazquez-Chona et al. (2005) have shown the genetic network in injured retina and found increased expression of *IER-3* gene with other transcription factors such as *Crem*, *Egr1, Fos, Fosl1, Junb, Egr1, Btg2, Atf3*, and* Nr4a1* *etc*. In addition to this, Gupta et al. (2005) have demonstrated the protein levels in human lens epithelial cells induced with glucocorticoid and found down regulation of *IER-3* gene in microarray analysis which was further confirmed by real time PCR. These genes are involved in angiogenesis and can exert their additive effect on AMD pathogenesis which may be independent to CFH mediated pathology.

Solute carrier family 16 member 8 (*SLC16A8*: 53kDA or Monocarboxylate transporter 3) is a transporter family works by proton motive force mediated by transportation of many monocarboxylates such as lactate. Pyruvate and branched-chain oxo-acids derived from Leu, Val, Ile, ketone bodies transport across cell membranes. Apart from transport function across plasma membrane, its role was also found in leucocytes migration and blood coagulation. Human RPE and choroids are the two retinal tissues where the expression of *MCT-3* was found abundantly. Among both, RPE expresses more amounts of *MCT-3* then choroid plexus epithelium in the basal of the cells (Philp et al., 2001).

Daniele et al. (2008) have reported the altered vision in *SLC16A8* knockout mice. *MCT3*^−^/^−^ mice have been shown to have lost expression of CD147, which form heteromer complex with *MCT-3*, from basolateral membrane of RPE but not from apical RPE resulting in reduced α-waves amplitude of scotopic electroretinogram and four folds increase in lactate in the retina consequently decreasing the pH of outer retina. In addition to this, Gallagher-Colombo et al. (2010) have demonstrated the decreased expression of *MCT-3* while healing of wound generated by scratch in chick RPE cells and human fetal (hf) RPE culture that may induce to pathologic phenomenon in the retina. The migrating cells at the site of wound demonstrated enhanced expression of *MCT4* and lacking the expression of MCT-3.

Recent study by Takeda et al. (2009) had shown *CCL-11* and *CCL-24* expression elevated in the mice treated with laser and further neutralization of both *CCL-11* and *CCL-24* with antibody significantly reducing the area of choroidal neovascularization (CNV) in the mouse retina, indicating its causal role in pathogenesis of AMD. Moreover, it has also been demonstrated that ciliary neutrophic factor (CNF) formation mediated by *Eotaxin*-*CCR-3* signaling, suggests the active role of* Eotaxin*-*CCR3* in choroidal endothelial cell proliferation in CNV formation which have been reduced by the administration of neutralizing antibody of *Eotaxin*-*CCR3*. Furthermore, the knockout mice of *eotaxin* and its receptor *CCR3* have been shown to have protective effect on laser induced CNV (Wang et al., 2011). Lately, it has been demonstrated that the angiogenic (Humbles et al., 2004) as well as allergic reactions in the body could be regulated by *eotaxin* with the involvement of mast and eosinophils (Salcedo et al., 2001). It was found that *eotaxin-CCR3* inhibition could regulate CNV formation strongly as compared to vascular endothelial growth factor (VEGF) regulation.

**Figure 1:**
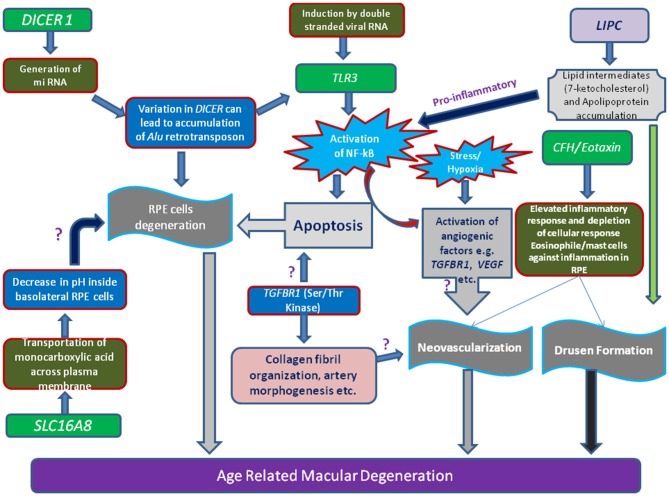
**Schematic representation of various genes loci and their linkage to AMD.** Illustration showing the toxicity of *Alu* miRNA and/or degraded retroviral RNA can activate of *NF-κ*B pathway mediated through Toll-like receptor 3 (*TLR3*) receptor. Stimulation of angiogenic (e.g., vascular endothelial growth factor, *VEGF*, Transforming growth factor-β receptor 1,*TGF*β*R1*), inflammatory and apoptotic pathways in retinal pigment epithelium (RPE) cells by oxidized lipid metabolites (e.g., 7-ketocholesterol) and apolipoproteins may signify the role of proteins (e.g., Lipase C, *LIPC*) involved in such processes. Additionally, increase of pH or concentration of monocarboxylic acid inside RPE cells which is regulated by transporter proteins (SLC16A8), can also hamper RPE cells function.

*DICER-1*, a multi-domain protein, is another important enzyme for synthesis of short interfering RNAs (siRNAs ~21–25 nucleotides) from pre-double-stranded RNAs during RNA interference. *DICER-1* is the miRNA processing enzyme that is required for the maturation of miRNAs. If there is any defect in the *DICER-1* then most of these RNAs cannot be generated. About 30% of the human genes are regulated by micro RNA classes of small RNA. However, mammalian *DICER-1* appears to play an important function in visual activities by degrading toxic RNA. It was recently reported that accumulation of transcripts of *Alu* RNA in the geographic AMD was caused due to dysregulation in the *DICER-1*. Kaneko et al. (2011) showed that the mice deficient in *DICER1* in RPE caused damage to the RPE similar to geographic atrophy. Mammalian *DICER-1* has also been recently reported to be involved in visual activities by degrading toxic RNA. It was reported that accumulation of transcripts of *Alu* RNA in the geographic AMD was caused due to dysregulation in the *DICER1* (Figure 1). Kaneko et al. (2011) showed that the levels of *DICER1* were found to be reduced in the advanced form of AMD i.e., geographic atrophy in AMD patient’s RPE.

In *SOD1*^−^^/−^ mice model, the role of *SOD1* which plays protective mechanism against nascent oxygen has already been reported. This impaired mechanism leads to the degenerative changes in the retina of *SOD1*^−^^/−^ mice. They have also shown cardinal features of AMD pathology in *SOD1*^−/–^ mouse retina like thickening of BM, CNV and drusen *etc* (Valentine et al., 2005). The adeno-associated virus (AAV) ribozyme mediated degradation of *SOD2* mRNA in RPE of the wild-type mice cause *SOD2* deficient mice (Justilien et al., 2007). This *SOD2* deficient mouse had BM and RPE changes and elevated level of A2E and lipofuscin accumulated in RPE (Kasahara et al., 2005). It has also been reported that the oxidative stress elevates the chance of the ocular neovascularization and can induce inflammation which lead to AMD. Several studies have demonstrated that significant association between genetic variation in *CFH* gene and AMD pathophysiology. In last few years, it has been found that genetic factors are strongly associated with incidence of AMD. Among these genetic factors, *CFH*, which is located on chromosome 1q32 and HtrA serine peptidase 1 (*HTRA1*), which is located on chromosome 10q26, are found more strongly associated with AMD. However, the molecular mechanisms need to be clarified. However, various studies have shown the interaction of *CFH* with *ARMS2* and *HTRA* (Huang et al., 2015) including smoking. Moreover, oxidized lipids also can show the higher affinity with *CFH* molecules which further can stimulate the macrophages to release the angiogenic (like *VEGF*) or proangiogenic molecules (like *IER*-3, *IL*-6/8; Sharma K. et al., 2014).

Genetic variants, especially complement system’s components, shows well-built association with AMD, defines the involvement of innate immune system. Therefore, the innate immune components, complement system and Toll-like receptor (*TLR*) which acts as pattern recognition (PRR) molecules for innate immune system (Kumar et al., 2004; Liu et al., 2008) function in response to their respective ligand in two ways: by stirring up the phagocytosis of target molecules and by activating the signal pathways that can provoke the expression of cytokines and other inflammatory mediators (Park et al., 2007; Van Beijnum et al., 2008). These augmented and sustainable inflammatory responses in RPE cells, by complement system, by *TLR* signaling, or by the co-activation of both, can stimulate the drusen formation in macula. Schröder and Bowie (2005) accounted for strong association between *TLR4* (a bacterial endotoxin receptor) variants and increased risk of AMD susceptibility. Yang et al. (2008) showed that *TLR3* variance rs3775291 plays a protective role in GA patients.

Various investigations have shown that the *CFH* gene on chromosome 1q31 is the first major AMD susceptibility gene (Zareparsi et al., 2005). The association of increased risk of AMD is reported to result from the Y402H variant in exon 9 (rs1061170, *T* > *C*, Sepp et al., 2006). *CFH* gene regulates the both alternative and classical complement pathways, therefore maintain the complement system mediated immunity. Any genetic variation in *CFH* gene which leads to structural or functional changes are believed to lead AMD pathology.

*VEGF* has been reported to be strongest angiogenic factor responsible for CNV in case of wet AMD pathology. A study of Taiwan, Chinese and English population has shown positive correlation between genetic variation of *VEGF-A* gene and AMD pathology (Churchill et al., 2006; Lin et al., 2008). On the contrary, Boekhoorn et al. (2008) did not reveal significant association in AMD pathology and polymorphisms of *VEGF* gene. Similarly, *VEGF* also plays an important role in AMD and corneal pathogenesis by inducing neovascularization (Philipp et al., 2000; Carneiro et al., 2011). *VEGF* binds to its three receptors viz., *VEGFR-1*, *VEGFR-2*, and *VEGFR-3*, and co-receptors such as neurophilin and heparan sulfate proteoglycans (Hiratsuka et al., 1998).

Transforming growth factor-β *(TGF-β)* cascade is another important signaling network that acts upon *TGFBR-1* and type II *TGF*-β receptors forming a heteromeric complex and transducing signal from the cell surface to the cytoplasm. TGF-β shows serine/threonine kinase activity and predominately expressed in highly active organs like heart and brain. Several important cellular functions are regulated and govern by TGF-β including apoptosis, conversion of epithelial to mesenchymal, hypoxic condition, artrial morphogenesis, transcriptional regulation, colagen fibril organization and signal transduction. Recently, meta-analysis by Fritsche et al. (2013) on GWAS has demonstrated 19 genetic loci which have shown the significant correlation with AMD pathology. In this study, *Collagen type XV* and *TGFBR-1* have found that both genes are located on chromosome 9q22.33. It has been reported that *type XV collagen* was found to have much expression in cells present at base and provide strength to vessels. The heterozygous mutation in either *TGFBR-1* or *TGFBR-2* was found to have new phenotypes which include perturbations in cardiovascular, skeletal development and neurocognitive systems. The expression levels of these proteins, higher in both collagen and connective tissue growth factors, with elevated level of phosphorylated *SMAD2* indicated increased *TGF* signaling despite not responding to *TGF* signaling in mutated alleles (Loeys et al., 2005). *VEGF*, basic fibroblastic growth factor-2 (*FGF2*), *TGFβ-1, 2, 3*, and *TGFBR-1*, 2, 3 are known as angiogenic factors. Enhanced mRNA levels were reported in tissue sample of cancer and neoplasia, and found elevated parallel to increased severity of disease as compared to that of normal cervical tissues (Soufla et al., 2005).

**Figure 2:**
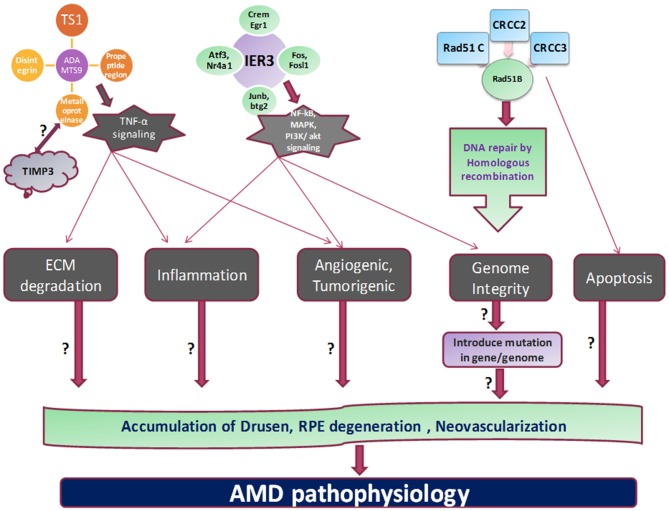
**Proposed mechanism and outstanding questions in AMD pathogenesis.** The various cellular functions like apoptosis, tumorigenesis, homologous recombination, angiogenesis, and inflammation are being regulated by different genes which propose to stimulate the cardinal feature of AMD pathology.

*RAD51* family members are involved in DNA repair generated during recombination and by DNA damaging agent by homologous recombination. *RAD51B* can regulate the several cellular activities by delaying the G1 cell cycle and inducing the apoptosis. These processes mediated by *RAD51B* could be induced by damage in DNA fragment (Figure 2). Three isoforms by alternative splicing of *RAD51B* have been identified yet. Various SNPs studies on *RAD51B* gene demonstrated important genetic factor contribute to breast cancer and colon cancer pathogenesis. Hamdy et al. (2011) revealed genetic variation in *RAD51B, RAD51* (G135C) and *XRCC3* (Thr241Met) genes found to be significantly correlated with leukemia pathology in cancer patients.

A disintegrin and metalloproteinase with thrombospondin motifs* (ADAMTS)* family proteins include various domains like thrombospondin type-1, metalloproteinase *etc*. These family proteins involve in proteolysis and several other biological processes like development and angiogenic processes. Bevitt et al. (2003) investigated the *ADAMTS* expression in RPE cells which are transcriptionally regulated by TNF-α signaling pathway. Mckie et al. (2003) have revealed the *ADAMTS* expression levels in response to TNF-α stimulation in ARPE-19 cells. Increased expression of *ADAMTS*1, *ADAMTS*6 and *ADAMTS*9 by TNF-α suggested their possible role in inflammatory conditions of retina. Moreover, expression of other *ADAMTS* in response to TNF-α also showed that *ADAMTS* families have their role in neovascularization, processing of collagen and cleavage of proteoglycan (Mckie et al., 2003). Expression regulation of ADAMTS family by TNF-α in retinal layers suggest the crucial role of these proteins in AMD and other inflammatory diseases.

Beta 1,3 glucosyltransferase (*B3GALTL*) encodes enzyme beta 1,3-galactosyltransferase-like which is located on chromosome number 13q12.3. This enzyme is involved in glycosylation of proteins. The *B3GALTL* gene is normally turned on in most cells of the body, which suggests that the B3Glc-T enzyme plays an important role in cellular function. Mutation in this gene cause eye disease known as Peter’s plus syndrome in front part of the eye called anterior chamber. Peter’s anomaly, characterized by attachment of cornea with iris and clouding of cornea, leads to blurred vision. This prominent mutation was discovered at c.597-2A > G position (located in CpG island). Bioinformatics analysis has revealed that this mutation causes the pre mRNA structural changes at secondary level. This mutation also suggests the role of epigenetic in modulation of secondary structure at mRNA level consequently can alter the expression of this protein and can also show the pleiotropic effect on several eye disorders (Siala et al., 2012).

Age-related maculopathy susceptibility 2 (*ARMS2*) gene is one of the genes which has widely been studied and replicated in several ethnic groups worldwide (Fuse et al., 2011). *ARMS2* location predominantly occurs at high energy demanding tissue like mitochondria-concentrated part of human photoreceptor cells. SNP variation in *ARMS2* genes could lead to reduced stability of the *ARMS2* mRNA (Fritsche et al., 2008).

*HTRA1* is serine proteases which is composed of four protein domains for binding of Insulin-like growth factor binding domain (ILGF), a kazal domain, a trypsin-like peptidase domain and a PDZ domain to accomplish different cellular functions. Recently, it has also been discovered by INDEYE study that polymorphisms in *ARMS2/HTRA1* locus are significantly associated with early and late AMD but instead of this locus the complement factors components like *C2, CFH* and complement factor B (*CFB*) have not shown positive correlation with AMD pathology (Sundaresan et al., 2012).

**Figure 3:**
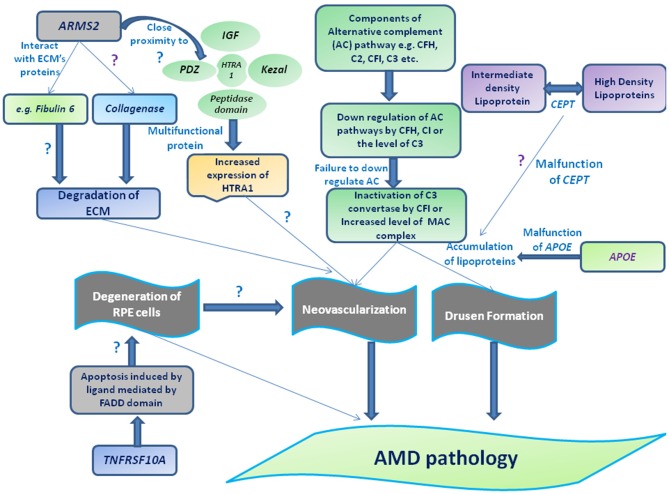
**Schematic representation of different genes and their association with AMD pathogenesis.** Drusen accumulation between RPE layers and Bruch’s membrane (BM) may be regulated through the components of alternative complement pathway (e.g., *CFH, complement factor I (CFI*), *C2* and *C3*) consequently leads to the formation of membrane attacking complex (MAC) and/or can also guide the choroidal neovascularization (CNV) by interacting with extracellularmatrix (ECM) proteins (e.g., *Fibulin 6, collagen*) or ECM maintenance proteins (*TIMP-3, ARMS2 or HTRA-ARMS2, collagenase etc*). Moreover, impaired function of *cholesteryl estertransfer protein (CETP*) and *APOE* may stimulate the deposition of apolipoproteins or other lipid metabolites assisting the formation of drusen along with complement factors or leading to degeneration of RPE by interacting apoptotic proteins or protein regulates apoptotic pathway (e.g. *tumor necrosis factor receptor superfamily 10A (TNFRSF10A) etc*).

Activation or dysregulation of several complement factors of alternative complements pathway like *CFH, C2*, complement factor I *(CFI)* and *CFB* have been found to be associated with AMD pathogenesis by releasing local inflammatory activating products. Several complement factor especially alternative complement pathway genes have been reported in pathogenesis of AMD (Figures 1, 3). Thakkinstian et al. (2012) pooled data from 19 studies which happened between 2006 and 2011 for 4 SNPs: rs9332739 and rs547154 for *C2* gene and rs4151667 and rs641153 for *CFB* gene and suggested the robust estimate that these alleles contributed to lowering the risk in all AMD pathogenesis in Caucasian population by 2.0% to 6.0%. Recently, it has been examined that complement factor B polymorphism (R32Q) greatly correlated with early AMD but have protective effect on late AMD in Caucasian population (Mantel et al., 2013).

Meta analysis based on GWAS by Holliday et al. (2013) have reported that *CFH* and *ARMS2* SNPs are significantly correlated with early AMD pathogenesis, and also suggested the polymorphisms of Apolipoprotein E (*APOE*) associated with early AMD out of many genes which have been studied in this study. This study also suggested that genetic variants were less effective in causing the pathogenesis of early AMD as compared to late AMD (Holliday et al., 2013).

Another gene cholesterylester transfer protein (*CETP*) is involved in reverse transfer of insoluble cholesteryl esters in the reverse transport of cholesterol and found to be associated with AMD pathogenesis. In 2010, a GWAS was conducted which reported an association of HDL with susceptibility towards AMD. The group conducted the study among 2157 cases and revealed strongest association signals of two genes namely *LIPC* and *CETP* with AMD (Chen et al., 2010). However, in recent case control study conducted in Chinese population having AMD (*n* = 535) *CETP* was studied as one of the gene among 10 genes having different variants. No significant association of *CETP* was shown in AMD patients. This might be possible that in case of genetic studies different genes might respond differently (Tian et al., 2012).

Tumor necrosis factor receptor superfamily 10A (*TNFRSF10A*) signaling plays a crucial role in apoptosis in cells. It has been identified as an important risk factor significantly correlated with AMD pathology. A GWAS carried out in 2011 among 1536 patients of AMD in Japanese population showed a significant association of disease susceptibility with *TNFRSF10A* gene on chromosome 8p21 (Arakawa et al., 2011).

Collagen is an important protein component of ECM and plays crucial role in cell-cell and cell-ECM adhesion. *COL10A1* is a gene for collagen protein and its normal function is important for neo vascularization. However, it is shown to be involved in risk factor for AMD disease. In 2011 study of genotypes of 2594 cases collectively studied the *VEGF* and *COL10A1*. This study with 2594 cases and 4134 controls recruitments. Total 6036699 SNPs from 1000 Genomes Project reference genotypes were analyzed and found two new common susceptibility alleles, near *FRK*/*COL10A1* and *VEGF-A* for AMD (Yu et al., 2011).

In all the above studies none have comprehensively examined the above loci or associated SNPs, their protein correlates and their role in AMD phenotype in a longitudinal fashion either in the Caucasian or Indian population.

Genotyping and evaluating associated variants may be more appropriate which can be analyzed by deriving a statistical model to find probable interactions and communication between complex set of genes. Hageman et al. (2011) have put forward a statistical model based on gene-gene interaction in AMD association studies and without accounting the environment factors like smoking, BMI, etc which might introduce inaccuracies in calculation. Moreover, Seddon et al. (2009) have analyzed unconditional logistic regression for genetic, ocular as well as environmental variables and the scores to discriminate progressor was found to be significant. Lately, Grassmann et al. (2012) have developed a statistical model to reveal the probable interaction between genetic variants and different environment factors. They have developed genetic risk score (GRS) for AMD for 13 genetic variants and exhibited good discriminative accuracy. In addition to AMD, the statistical modeling in other diseases have been established and validated. We have previously validated the Hosmer–Lemeshow goodness of fit statistic and provide binary logistic regression model in Amyotrophic Lateral Sclorosis (ALS) pathology (Gupta et al., 2012). Therefore, for better assessment of genes interaction and regulation with or without including environmental factors can be analyzed by statistical modeling which can open a new vista in diagnostic field and management of AMD pathology.

Hence, in addition to statistical modeling, using computational approach, we can analyze the effect of nsSNPs observed in the candidate genes in the AMD patients. We can map nsSNPs onto the biologically significant features of proteins such as domains, families, folds, post-translational modifications (PTMs) and protein sorting motifs. These features are known to play important role in predicting the protein function. Thus, it can provide an insight how the changes at genomic level reflects at the protein level, affecting protein function and leading to the progression of AMD. The study of nsSNPs in candidate genes provides better understanding of the phenotype variation in North Indian AMD patients. Therefore, the statistical modeling and bioinformatics analysis would greatly strengthen the outcome of AMD research as it would enable prediction of probable interaction between different genes variants and risk factors and structural, functional changes in proteins respectively.

AMD is a heterogenic and multi-factorial disease which can also be influenced by environmental factors. We can address the missing genetic links and examine the probable interaction between gene-gene and gene-protein by bioinformatics analysis and can map the mechanistic network. Such genetic studies can provide probable diagnostic/prognostic markers and new therapeutic targets for the disease by selecting common or novel molecules from such genetic network.

The predictive interactions and statistical modeling of various variants analyzed by GWAS could provide the bigger mechanistic canvas at the genomic level. Therefore, we can identify new target therapy in such complex heterogenic disease (for both avastin responder and non-responders) and/or can also assist the prevailing therapies (like anti-VEGF therapies) based on individual genetic interactions.

## Statistical Analysis and Modeling

Mendelian randomization analysis on epidemiological studies has been shown to remove many potential biases and confounding factors. It helps in increasing the robustness of data (Thomas and Conti, 2004). Mendelian randomization is a technique based on second principle of Mendelian genetics *i.e.*, the law of random assortment which suggests two traits can be inherited in the progeny independent to each other (Bochud and Rousson, 2010). In conventional studies the association of genetic and phenotype variation is used to describe the gene function, and the mode of assessing the genetic variation is done by analyzing the SNPs that are sufficient and frequent in a population for making significant comparisons. Mendelian randomization uses genetic epidemiology to make causal inference of a disease (Smith and Ebrahim, 2003; Lawlor et al., 2008). Mendelian randomization is an application of Instrument variables (IV) and thus uses genetic information as its IVs (Valentine et al., 2005). Formally, an IV is a variable/genotype (*Z*) that follows a set of conditions. The outcome of *Z* is correlated with the exposure to be analyzed and its independent value not associated with confounding factors (U) effects. Therefore, *Z* is independent results of *X* and *Y* which shows the association between *Y*, *X* and its confounding factors (U) (Thomas and Conti, 2004).

However, linkage disequilibrium (LD) approach has been widely used in the several studies that show the association among genetic alleles in a set of population to discover the genetic markers. LD and pleiotrophic are the two conditions which can be used to identify and understand the function of specific gene variant in homogeneous population. These genetic variants could show the pleiotrophic effect on expression of that or other associated genes via involvement of various biological pathways in presence of modifiable exposure (environmental factors). These could be used to analyze the biological interaction between genetic variants and modifiable factors (Thomas and Conti, 2004). Therefore, LD can show the interaction between the selected genotype and the polymorphism then the association of modifiable exposure risk factors to outcomes may be confounded (Thomas and Conti, 2004).

Typically, an equation representing the variables can be derived as:

For *p* variant size observed lets assume *“n”* subjects from a region are sequenced. Age, gender, and SNP variants could be included as covariates for population stratification. Hence, phenotype variable for *i-*th subject can be represented as:

$$X_{i}\;=\;(X_{i1},X_{i2},\ldots ,X_{im})$$

and genotype model can be represented as

$$G_{i}\;=\;(G_{i1},G_{i2},\ldots ,G_{ip})$$

Lets assume 0, 1, or 2 the copy number of minor alleles present in genetic analysis of set of genes which could show the additive effect on disease phenotype. Logistic model could be represented as:

$$\text{logit}\left[P(y_{i}\;=\;1)\right]\;=\;\alpha_{0}+\alpha \prime X_{i}+\beta \prime G_{i}$$

For SKAT analysis on same set of genes in a population and their correlation with environmental factors could be compute by assuming the null hypothesis as H_0_: *β* = 0, that is, *β_1_* = *β_2_* = … = *β_p_* = 0, so model can be represented as:

$$Q\;=\;{\left(y-\hat{\mu }\right)}^{\prime }K\left(y-\hat{\mu }\right)$$

Here *K* is Karnal function.

To correlate genotypes and phenotypes, including effects of covariates like age, sex etc, one may carry out SKAT. This procedure is available in R software as well as in SAS (Statistical Analysis Software). Marginal effects in gene interaction and correlation can also be obtained by using General Linear Model (GLM), available in SPSS (Statistical Product and Service Solutions).

The effect of confounders on genetic variants and its role could be explained by statistical modeling like Mendelian randomization, logistic modeling (which takes care of categorical variables like smoker/non-smoker etc.) and SKAT analysis. Epistasis phenomenon has been shown in various studies to detect the effect of different genes interaction with *CFH* and AMD phenotypes (Maller et al., 2006). Apart from epistasis phenomenon, additive effect could also be seen amongst various genes and their role in advancement of AMD pathology.

## Bioinformatic Analysis of SNPs in Genes

**Figure 4:**
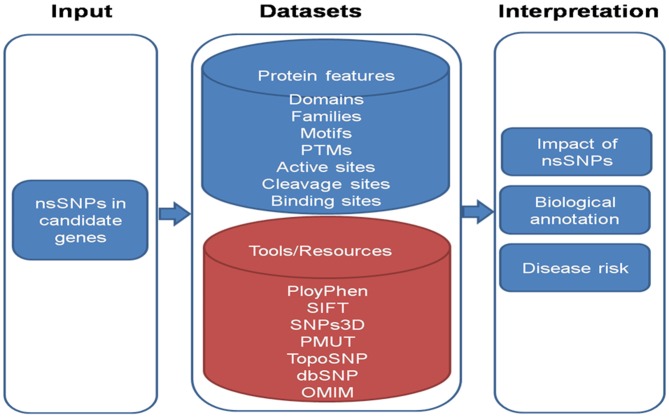
**A schematic overview of the work flow to analyze nsSNPs**.

To analyze the effect of snSNP of genes we can employ following workflow as shown in Figure 4. The information of various domain, families and motifs like protein sorting motifs, coiled-coil motifs and regulatory motifs could be matched with existing database. Important amino acid residues in protein sequences which include PTMs (post translational motifs), active sites, cleavage sites and binding sites can also be included in the dataset. These features are known to play important role in determining the protein function. These protein features can be taken from: (i) Human Protein Reference Database (HPRD) [PMID: 18988627], which contains manually curated information pertaining to the biology of human proteins; and (ii) Swiss-Prot database, which comprises of highly curated information about proteins. In-house Python scripts can be used to map the nsSNPs onto significant protein features.

To interpret the impact of nsSNPs, in-house python scripts can be used to look for conservative/non-conservative nsSNPs across classes of amino acids and change of nsSNP to cysteine and *vice versa*. Evolutionary conservation of nsSNPs across multiple species including *Macaca mulatta, Pan troglodytes, Mus musculus, Rattus norvigicus, Bos taurus, Canis lupus familiaris* (dog), *Equus caballus* (horse), *Gallus gallus* (chicken) and *Danio rerio* can be studied. Local alignment can be generated encompassing variant using European Molecular Biology Open Software Suite (EMBOSS) and conservation of variant could be checked using in-house Python scripts.

Further, available tools for the prediction of biological effect of nsSNPs such as polymorphism phenotyping-2 (PolyPhen -2) [PMID: 20354512] considers sequence and structural feature of proteins; Sorting Intolerant From Tolerant [SIFT; PMID: 12824425] is based on evolutionary conservation of protein sequences; SNPs3D [PMID: 16551372] is based on protein sequence conservation, protein structure and stability; PMUT [PMID: 15879453] scans mutational hot spots in three-dimensional structure of proteins and TopoSNP [PMID: 14681472] uses geometric location of nsSNPs in three- dimensional structure of protein, can be used. Variant amino acid residue can be searched via Online Mendelian Inheritance in Man (OMIM) database in order to infer nsSNPs to be damaging.

Novel changes can be evaluated for their potential effect on the protein structure and function using bioinformatics tools. Together these approaches provide a useful tool which can be very useful in describing the AMD spectrum among Indians.

## Conclusion

AMD is a genetically heterogeneous disease which could be equally influenced with environmental factors. Most of population/epidemiological studies in AMD are confined up to the genetic analysis of various genes and their association with pathology progression. Here, we tried to describe the integrative approach in AMD genetics by combining demographic and genetic data, and thus ultimately can propose statistical model for the same for better management of disease prognosis and diagnostic field. Bio-informatics approach could also assist the nature of SNP variant by analysing the impact of SNP changes in the structural and functional aspect of the particular protein.

## Author Contributions

AA: conceptualization, editing and writing of the manuscript, KS: assistance in conceptualization and writing of manuscript, SKS: statistical modeling and Mendelian randomization concept, RS: editing of manuscript, NKS: editing of manuscript; KP: bioinformatics concept in the manuscript.

## Conflict of Interest Statement

The authors declare that the research was conducted in the absence of any commercial or financial relationships that could be construed as a potential conflict of interest.
